# How to involve ‘hard to reach’ population groups in research projects: Lilian van der Ven

**DOI:** 10.1093/eurpub/ckac129.476

**Published:** 2022-10-25

**Authors:** LGL van der Ven, JM van Baar, RS Wispelweij, M Jambroes

**Affiliations:** 1 Julius Center for Health Sciences and Primary Care, University Medical Center Utrecht, Utrecht University, Utrecht, Netherlands; 2 Trimbos Institute, Utrecht, Netherlands

## Abstract

**Issue:**

Addressing and reducing health inequalities is a major challenge in public health. Actively involving the most vulnerable populations groups in research projects in order to assess and address their needs is an important step towards reducing health inequalities. However, the most vulnerable groups are often perceived as the most difficult to reach for researchers.

**Description:**

We conducted a research project to assess and address the health effects of the COVID-19 pandemic in vulnerable population groups. In three neighbourhoods, we identified the 2-3 most vulnerable population groups. We aimed to interview at least 20 participants per neighbourhood, evenly distributed among the groups, using creative and adaptive recruitment methods.

**Results:**

Successful strategies differed per group. Strategies that proved successful were: rewriting information and consent letters together with the target population to help make them more accessible and attractive; using simple language in texts and while interviewing; asking the target population what type of compensation for the interview they would find most attractive; identifying persons who function as social hubs in the neighbourhood, asking them to help recruit participants, and providing them with financial compensation for their time; asking professionals in care and welfare organisations to help recruit among their clients; interviewing people in the places they would normally go to; snowball sampling. We met our recruitment goals.

**Lessons:**

When working with ‘hard to reach’ populations, it takes researchers time, flexibility and creativity to find the right strategy to recruit participants while still respecting the boundaries set by GDPR and ethics committees. We recommend researchers to think about various possible recruitment strategies before starting a project, and to be prepared to change strategies during a project if necessary.

**Key messages:**

• ‘Hard to reach’ populations are not really hard to reach, they just require a different mindset and skillset from the researcher.

• We encourage researchers to be creative and flexible in finding the right recruitment strategies in their project, and to think about various possible strategies before starting a project.

